# A prospective cohort study of the impact of chronic disease on fall injuries in middle-aged and older adults

**DOI:** 10.1515/med-2023-0748

**Published:** 2023-07-13

**Authors:** Xue Yang, Longxin Li, Fang Xie, Zhang Wang

**Affiliations:** Department of Geriatrics, The General Hospital of Western Theater Command, Chengdu, P.R. China

**Keywords:** chronic disease, fall injury, risk factors, health management, cohort studies

## Abstract

This cohort study investigated the impact of chronic diseases on fall risk in middle-aged and older individuals, offering insights for fall prevention strategies. Analysing data from 4,670 participants aged 40+ years, we used a Cox proportional risk model to assess chronic disease types, numbers, and interactions with other factors on fall injury risk across age groups. Results showed that middle-aged adults with respiratory diseases had a 26% increased fall risk (hazard ratio [HR] = 1.26, 95% confidence interval [CI]: 1.05–1.48), and a linear dose–response relationship was observed between chronic disease number and fall risk (*p* < 0.001). The study also examined interaction effects of chronic diseases with gender, disability, and fall injury history. Female middle-aged and older adults with chronic diseases had a 67% higher fall risk than their male counterparts without chronic diseases (HR = 1.67, 95% CI: 1.36–1.88). In conclusion, chronically ill middle-aged and older adults have a higher fall risk, with high-risk groups including women, those with chronic diseases, and individuals with fall injury history. Fall prevention efforts should target middle-aged adults as well.

## 1 Introduction

An estimated 36.89 million fall-related injuries occur annually across the globe. Numerous chronic conditions, such as arthritis, diabetes, and cardiovascular disease, are believed to increase the risk of fall-related injuries [1,2]. Older adults are a high prevalence group for chronic diseases, and falls in older adults are frequently associated with poor prognosis and high rates of disability, making them susceptible to serious injury and thus a widespread concern [3]. In China, falls account for more than 46% of unintentional injuries among the elderly and are the leading cause of injury-related deaths among the elderly [4,5]. In recent years, chronic diseases have shown a younger trend. The prevalence of chronic diseases in the middle-aged population aged 40–59 years in China has reached 61.24%, and the proportion of co-occurring chronic diseases accounts for 51.98% of the middle-aged population [6,7]. The study by Kistler et al. that the current disease prevention window moving forward has become a new paradigm for health management [8]. It is equally important to explore the impact of chronic disease on the recent and long-term occurrence of fall injuries in middle-aged people [9].

Nationally and internationally, research on the association between chronic disease and fall injuries is well documented [10,11]. However, most studies in China have explored the association using mainly cross-sectional data, and few cohort studies have focused on the effect of chronic disease on fall injury [12]. There is little literature discussing the impact of co-occurring chronic conditions and the interaction of chronic conditions with other risk factors on the risk of fall injury [13,14]. However, the coexistence of co-occurring chronic diseases, chronic diseases and other fall risk factors has become a significant problem for people at high risk of falls today [15]. This study used data from the elderly follow-up survey conducted by the Fifth People’s Hospital of Sichuan Province to investigate the impact of chronic diseases on fall injuries in the middle-aged and elderly population using a prospective cohort study design and to provide data support for future studies focusing on the prevention and reduction of fall injuries in middle-aged and elderly individuals.

## 2 Patients and methods

### 2.1 Data sources

The elderly follow-up survey conducted by the Fifth People’s Hospital of Sichuan Province mainly collected data from middle-aged and elderly people in 28 provinces in China, covering demographic information and health status information, and the sampling design and data quality were assessed. The baseline survey for this project started in 2012, and the follow-up survey will be conducted until 2020. A total of 6,043 eligible middle-aged and older adults ≥40 years old recruited from the 2012 baseline sample were used for the study. A total of 1,126 people withdrew from the follow-up survey due to death or loss to follow-up, and 4,917 were reported at the end of follow-up; 247 respondents with missing variables or low questionnaire reliability and validity were excluded, resulting in the inclusion of a total valid sample of 4,670 people with a mean age of 58.52 years. The difference between the frequency of middle-aged and older adults in the pre- and postsample exclusion cohorts who experienced a fall injury and those who did not was not statistically significant (χ^2^ = 0.598, *p* = 0.537), indicating that selection bias due to data cleaning was less likely. Therefore, the sample size used to analyse the impact of baseline exposure factors on follow-up outcomes in this study was 4,670 cases.


**Ethics approval and consent to participate:** The patient in our research has signed the informed consent. This study was designed in accordance with the Declaration of Helsinki and approved by the Ethics Committee of the Fifth People’s Hospital of Sichuan Province, approval number: 20191209871624.
**Consent for publication:** Informed consent was obtained from all patients included in this study prior to the submission of data.

### 2.2 Baseline survey

A prospective cohort study design was used, with 4,670 middle-aged and older adults aged ≥40 years surveyed in 2012 as the baseline population. Information on exposure factors collected from the baseline survey was divided into two categories: study factors and confounding factors.

(1) The study factors are chronic disease prevalence, including cardiovascular disease (hypertension and heart disease), respiratory disease (asthma, chronic bronchitis, and emphysema), digestive disease (stomach disease and liver disease), metabolic disease (diabetes and dyslipidaemia), neurological disease (Parkinson’s disease and stroke), arthritis or rheumatism, and kidney disease.
(2) Confounding factors: These included demographic factors (gender and literacy), lifestyle factors that were more strongly associated with the risk of fall injury (length of lunch break and length of night sleep), and individual health status factors (ability to perform activities of daily living [ADL], physical pain, depressive symptoms and history of fall injury), where age was adjusted for as a stratifying factor. Both study factors and confounding factors were collected by questionnaire. Chronic disease was a condition for which a definitive diagnosis was made by the healthcare facility where the patient was interviewed. This cohort’s prevalence of chronic illnesses among middle-aged and elderly individuals is comparable to other national findings. Due to the prevalence of chronic illness co-morbidity among middle-aged and older persons, this study investigated the strength of the connection between the number of chronic diseases and the risk of injury from falls by defining the number of chronic diseases as 0, 1, 2, and ≥3 chronic diseases. Among the confounding factors, ADL and depressive symptoms were measured by the ADL scale and the Centre for Epidemiological Studies Depression Scale (CES-D) developed by Gazibara et al. [16].

The Kistler et al.’s study showed that the ADL scale and CES-D have high reliability and validity in reflecting the mental and health status of older Chinese people [17]. A history of injury from falls is widely regarded as an important risk factor in fall injury risk studies. In this study, a history of falls was defined as the occurrence of a serious fall injury in the past 2 years (2010–2011) at the baseline survey point, with severity defined by the need for medical treatment. The reason for defining fall injury history in terms of medical treatment for falls is that moderate-to-severe falls tend to cause a decline in an individual’s physical, psychological, and social functional status, which is of greater concern and reduces recall bias.

### 2.3 Follow-up survey

The middle-aged and elderly cohorts were followed up for fall injuries in 2014, 2017, and 2020. The outcome events obtained at the end of follow-up were categorised as “occurrence of fall injury” and “no occurrence of fall injury.” A fall injury event was defined as a fall visit from 2011 to 2018, and a fall injury was defined as not occurring if no fall injury had occurred by the end of follow-up. Due to the significant health heterogeneity between middle-aged and elderly people, the study population was divided into two groups by age stratification to analyse the effect of chronic diseases on the risk of fall injury in different age groups.

### 2.4 Statistical analysis

R 4.0 software was employed for data cleansing and statistical analysis. The life table method was used to calculate fall injury reporting rates for middle-aged adults aged 40–59 years, older adults aged ≥60 years, and the full sample population during the follow-up period; descriptive statistics on baseline exposure characteristics of the sample population by the occurrence of fall injury and non-occurrence of fall injury event outcomes, with *n* (%) representing the distribution of categorical variables, and log-rank one-way tests were used to analyse the relationship between exposure factors and middle-aged and older adults. A Cox proportional risk model was used to evaluate the strength of the association between the various categories of chronic illnesses, the number of chronic diseases, and the risk of fall injury in the middle-aged and elderly population, and to determine the hazard ratio (HR) and its 95% confidence interval (CI). Finally, an additive model was utilised to examine the effect of the interaction between chronic diseases and other exposure factors on the likelihood of falls and injuries in middle-aged and elderly individuals. Two-sided test was conducted for inferential statistics, and *p* < 0.05 was considered statistically significant.

## 3 Results

### 3.1 Reporting rate of fall injuries

As shown in Table 1, using the 2,012 baseline survey as a starting point, the fall injury reporting rates for the 4,670 study participants at 2, 5, and 7 years of follow-up were 7.98% (95% CI: 7.64–8.26%), 14.63% (95% CI: 13.52–15.03%), and 27.11% (95% CI: 26.24–29.68%), respectively. The three-time follow-up fall injury reporting rates for the 2,573 middle-aged adults were 7.19% (95% CI: 6.91–7.43%), 13.52% (95% CI: 12.84–14.18%), and 19.27% (95% CI: 18.79–19.64%), respectively. The three-time fall injury reporting rates for the 2,097 older adults were 8.96% (95% CI: 8.58–9.31%), 15.16% (95% CI: 14.86–15.53%), and 35.19% (95% CI: 34.99–35.86%), respectively. indicating that close to 1/6 of middle-aged adults aged 40–59 years and more than 1/3 of older adults ≥60 years had a fall injury event within 8 years of follow-up, respectively.

**Table 1:** Reporting rate of fall injuries during the follow-up period for 4,560 middle-aged and elderly people

Survey year	40–59 years old (injuries/effective sample)	≥60 years old (injuries/effective sample)	Total fall injury reporting rate (95% CI)
Baseline	/2,573	/2,097	/
2012			
2014	185/2,573	188/2,097	7.98 (7.64–8.26)
2017	348/2,573	318/2,097	14.63 (13.52–15.03)
2020	496/2,573	742/2,097	27.11 (26.24–29.68)

### 3.2 Results of the one-way analysis

The baseline characteristics are shown in Table 2, where gender, education, ADL, physical pain, depressive symptoms, history of fall injury, cardiovascular disease, respiratory disease, digestive disease, arthritis, renal disease, length of lunch break, and length of sleep were statistically associated with the risk of fall injury in middle-aged adults (*p* < 0.01); gender, education, ADL, physical. There was a statistical association between gender, education level, ADL, physical pain, depressive symptoms, history of fall injury, digestive disorders, arthritis, renal disorders, length of lunch break, length of sleep, and risk of fall injury in older adults (*p* < 0.01). There was a statistical link between the remaining exposure variables and risk of fall injury in middle-aged and older persons; however, there was no statistical association between neurological and metabolic illnesses and risk of fall injury (*p* < 0.01). The other exposure variables were statistically linked with the risk of fall injury in adults aged middle-aged and older (*p* < 0.01).

**Table 2:** Baseline characteristics of respondents and log-rank one-way test

Variables	40–59 years old (*n* = 2,573)			≥60 years old (*n* = 2,094)			Total (*n* = 4,670)		
	Injuries falls (*n* = 496)	No fall injuries (*n* = 2,077)	*p* value	Injuries from falls (*n* = 742)	No fall injuries (*n* = 1,352)	*p* value	Injuries from falls (*n* = 1,238)	No fall injuries (*n* = 3,432)	*p* value
**Sex**			<0.001			<0.001			<0.001
Male	168	872		296	621		464	1,493	
Female	328	1,205		446	731		774	1,936	
**Education level**			<0.001			<0.001	0	0	<0.001
Primary school and below	132	358		306	518		438	876	
Junior high school	149	825		323	604		472	1,429	
High school and above	115	894		113	230		228	1,124	
**Ability to perform ADL**			<0.001			<0.001	0	0	<0.001
Yes	422	2,036		685	1,249		1,107	3,285	
No	74	41		57	103		131	144	
**Physical pain**			<0.001			<0.001	0	0	<0.001
No	266	1,629		434	894		700	2,523	
Yes	230	448		308	458		538	906	
**Depressive symptoms**			<0.001			<0.001	0	0	<0.001
No	304	1,447		399	958		703	2,405	
Yes	192	630		343	394		535	1,024	
**History of injuries from falls**			<0.001			<0.001	0	0	<0.001
No	413	1,894		618	1,217		1,031	3,111	
Yes	83	183		124	135		207	318	
**Cardiovascular disease**			0.011			0.006	0	0	<0.001
No	319	1,752		383	958		702	2,710	
Yes	177	325		359	394		536	719	
**Respiratory diseases**			<0.001			0.157	0	0	<0.001
No	431	1,961		689	1,186		1,120	3,147	
Yes	65	116		53	166		118	282	
**Digestive system diseases**			<0.001			0.007	0	0	<0.001
No	299	1,594		509	984		808	2,578	
Yes	197	483		233	368		430	851	
**Metabolic system diseases**			0.462			0.571	0	0	0.898
No	415	1,828		677	1,104		1,092	2,932	
Yes	81	249		65	248		146	497	
**Neurological disorders**			0.358			0.452	0	0	0.406
No	471	2,024		697	1,281		1,168	3,305	
Yes	25	53		45	71		70	124	
**Arthritis**			<0.001			<0.001	0	0	<0.001
No	329	1,542		384	905		713	2,447	
Yes	167	535		358	447		525	982	
**Kidney disease**			<0.001			0.001	0	0	<0.001
No	443	1,993		669	1,194		1,112	3,187	
Yes	53	84		73	158		126	242	
**Length of naps (min)**			<0.001			<0.001	0	0	<0.001
No naps	281	760		404	684		685	1,444	
<30	57	475		85	167		142	642	
≥30	158	842		253	501		411	1,343	
**Length of sleep (h)**			<0.001	742	1,352	<0.001	742	1,352	<0.001
<5	113	544		261	65		374	609	
5–8	181	704		234	528		415	1,232	
≥8	202	829		247	759		449	1,588	

### 3.3 The impact of different chronic diseases on the risk of injury from falls

As shown in Table 3, after adjusting for the confounding effects of gender, education, ADL, physical pain, depressive symptoms, history of fall injury, length of lunch break, and length of sleep, the results of the Cox proportional risk regression model for the group of middle-aged adults aged 40–59 years showed that compared with middle-aged adults without respiratory disease, having respiratory disease increased the risk of fall injury by 26% (HR = 1.26, 95% CI: 1.05–1.48), and arthritis increased the risk of fall injury by 38% (HR = 1.38, 95% CI: 1.29–1.48). Cox proportional risk regression models for the group of older adults aged ≥60 years showed that arthritis increased the risk of fall injury by 21% (HR = 1.21, 95% CI: 1.16–1.28), and kidney disease increased the risk of injury from falls by 31% (HR = 1.31, 95% CI: 1.12–1.47). The results of the Cox proportional risk regression model for the middle-aged and elderly groups showed that having respiratory disease increased the risk of injury from falls by 19% (HR = 1.19, 95% CI: 1.11–1.32), having arthritis increased the risk of injury from falls by 28% (HR = 1.28, 95% CI: 1.16–1.37), and having kidney disease increased the risk of injury from falls by 24% (HR = 1.24, 95% CI: 1.14–1.37).

**Table 3:** A Cox proportional risk regression model of the effect of different chronic conditions on the risk of injury from falls in middle-aged and older adults

Variables	40–59 years old	40–59 years old	Total
**Cardiovascular disease**			
No	1.00	1.00	1.00
Yes	1.04 (0.89–1.17)	1.08 (0.95–1.19)	1.06 (0.92–1.16)
**Respiratory diseases**			
No	1.00	1.00	1.00
Yes	1.28 (1.12–1.39)	1.05 (0.96–1.13)	1.19 (1.11–1.32)
**Digestive system diseases**			
No	1.00	1.00	1.00
Yes	1.06 (1.01–1.20)	0.98 (0.91–1.06)	1.02 (0.99–1.08)
**Neurological disorders**			
No	1.00	1.00	1.00
Yes	0.86 (0.71–1.02)	1.09 (0.89–1.18)	0.97 (0.94–1.06)
**Arthritis**			
No	1.00	1.00	1.00
Yes	1.38 (1.29–1.48)	1.21 (1.16–1.28)	1.28 (1.16–1.37)
**Kidney disease**			
No	1.00	1.00	1.00
Yes	1.08 (1.01–1.15)	1.31 (1.12–1.47)	1.24 (1.14–1.37)

### 3.4 The effect of the number of chronic diseases on the risk of injury from falls

The results of the Cox proportional risk regression model are shown in Table 4. After adjusting for confounders, having 1, 2, and ≥3 chronic conditions increased the risk of fall injury in middle-aged and older adults by 29% (HR = 1.31, 95% CI: 1.20–1.39), 39% (HR = 1.39, 95% CI: 1.26–1.51) and 46% (HR = 1.46, 95% CI: 1.37–1.63), respectively. By age group, having 1, 2, and ≥3 chronic conditions increased the risk of fall injury by 35, 47, and 54% in middle-aged adults aged 40–59 years, respectively; older adults aged ≥60 years with ≥3 chronic conditions had a 31% higher risk of fall injury than those without chronic conditions (HR = 1.31, 95% CI: 1.24–1.38). The trend test showed that the association between the number of chronic conditions and the risk of fall injury was a dose‒response relationship in the middle-aged, elderly, and combined middle-aged and elderly groups, indicating that the higher the number of chronic conditions in middle-aged and elderly people, the higher the risk of fall injury. The overall χ^2^ value of the trend test was further decomposed into a linear regression component and a partial linear regression component, and it was found that the linear regression component was statistically significant in the middle-aged and elderly groups, while the partial linear regression component was not statistically significant, and both components were statistically significant after age grouping, indicating a linear relationship between the number of chronic diseases and the risk of fall injury in the middle-aged and elderly groups.

**Table 4:** Dose–response relationship between the number of chronic diseases and the risk of injury from falls in middle-aged and elderly people

Model A	40–59 years old (*n* = 2,573)	≥60 years old (*n* = 2,097)	Total (*n* = 4,670)
**Number of chronic diseases**			
0	1.00	1.00	1.00
1	1.35 (1.27–1.51)	1.21 (1.10–1.32)	1.29 (1.20–1.39)
2	1.47 (1.31–1.59)	1.25 (1.14–1.43)	1.39 (1.26–1.51)
≥3	1.54 (1.44–1.68)	1.31 (1.24–1.38)	1.46 (1.37–1.63)
**Trend test**			
χ^2^ value	78.62	51.04	129.78
*p* value	<0.001	<0.001	<0.001
Pr value	<0.001	<0.001	<0.001
Pb value	0.019	0.008	0.329

### 3.5 The effect of chronic disease interacting with other factors on the risk of injury from falls

As shown in Table 5, after adjusting for confounders, the risk of fall injury was 67% higher for female middle-aged and older adults with chronic disease than for male middle-aged and older adults without chronic disease (HR = 1.67, 95% CI: 1.36–1.88), 68% higher for disabled middle-aged and older adults with chronic disease than for non-disabled middle-aged and older adults without chronic disease (HR = 1.68, 95% CI: 1.43–1.81), and middle-aged and older adults with a history of chronic disease and fall injury had a 162% higher risk of fall injury than middle-aged and older adults without a history of chronic disease and fall injury (HR = 2.62, 95% CI: 2.51–2.94). Female middle-aged adults with chronic disease had a 61% greater risk of fall injury than male middle-aged adults without chronic disease, while disabled middle-aged adults with chronic disease had a 54% greater risk of fall injury than middle-aged adults without chronic disease and non-disabled adults, and middle-aged adults with a history of chronic disease and fall injury had a 189% higher risk of fall injury than middle-aged adults without a history of chronic disease and fall injury. Similarly, the interaction of having a chronic disease with other exposure factors increased the risk of fall injury in older adults by 78, 76, and 139%, respectively.

**Table 5:** The effect of the interaction of chronic disease and other factors on the risk of injury from falls in middle-aged and older people

Models		40–59 years old (*n* = 2,573)	≥60 years old (*n* = 2,097)	Total (*n* = 4,670)
**Model 1**				
Chronic diseases	Sex			
No	Male	1.00	1.00	1.00
Yes	Male	1.19 (1.12–1.39)	1.07 (1.03–1.28)	1.13 (1.07–1.35)
No	Female	1.17 (1.08–1.32)	1.39 (1.16–1.52)	1.26 (1.12–1.44)
Yes	Female	1.61 (1.42–1.78)	1.78 (1.51–1.96)	1.67 (1.36–1.88)
**Model 2**				
Chronic diseases	Ability to perform ADL			
No	Yes	1.00	1.00	1.00
Yes	Yes	1.29 (1.20–1.36)	1.22 (1.17–1.49)	1.26 (1.24–1.38)
No	No	0.94 (0.79–1.07)	1.62 (1.14–1.98)	1.25 (0.86–1.73)
Yes	No	1.54 (1.21–1.76)	1.76 (1.52–2.10)	1.68 (1.43–1.81)
**Model 3**				
Chronic diseases	History of injuries from falls			
No	No	1.00	1.00	1.00
Yes	No	1.40 (1.31–1.64)	1.21 (1.13–1.49)	1.33 (1.24–1.36)
No	Yes	2.94 (2.45–3.70)	1.69 (1.32–1.98)	2.17 (1.94–2.69)
Yes	Yes	2.89 (2.51–3.06)	2.39 (1.97–2.84)	2.62 (2.51–2.94)

## 4 Discussion

This study indicated that the incidence of fall injuries was significantly higher in older people aged 60 years than in middle-aged people aged 40–59 years [18]; nonetheless, with the trend towards younger age groups with chronic diseases, middle-aged people also face a public health issue of a higher risk of fall injuries due to multiple chronic diseases [19,20]. This study revealed that both the type and number of chronic diseases had a significant effect on the incidence of fall injuries, with arthritis, renal and respiratory diseases, and comorbidities being independent risk factors for fall injuries in middle-aged and older adults, and that women, people with ADL impairment, and a history of fall injuries in middle-aged and older adults with chronic diseases were at high risk for fall injuries.

Hoffman et al.’s study showed that having arthritis leads to chronic motor dysfunction in older people, which increases the risk of fall injuries [21]. This study shows that middle-aged patients with arthritis also need attention to prevent fall injuries, which is consistent with Hoffman et al.’s study. A meta-analysis of fall injuries in adults with arthritis showed that impaired balance, muscle weakness, increased comorbidity, and knee pain were all risk factors for fall injuries in adults. In terms of the biomechanical and sports medicine processes involved in falls, patients with knee osteoarthritis often need to compensate for reduced weight-bearing capacity by prolonging lower limb support, reducing stride length, and increasing stride width when walking or exercising, resulting in greater reliance on hip abductors and extensors and difficulties in weight transfer, resulting in increased mechanical stress on the knee joint and disruption of body balance [22,23]. The immunopathogenesis of rheumatoid arthritis is not yet fully understood. However, research indicates that the arthritic process is accompanied by the ageing of muscle function, including bone fragility, loss of cartilage flexibility, deterioration of muscle strength, and loss of ligament flexibility, which all increase the risk of fall injury [24,25].

This study found that both kidney disease and respiratory disease were associated with an increased risk of fall injury in middle-aged and older adults. A cross-sectional study by Rai et al. [26] found no association between kidney disease and fall injury risk after adjusting for potential confounders but acknowledged that fall injury is a common problem in older people with kidney disease. As renal disease is often complicated by health problems, such as anaemia, disorders of calcium and phosphorus metabolism, and hypoproteinaemia, as renal function declines, predisposing to dizziness and weakness, malnutrition, and osteoporosis, the results of this study support the use of renal disease as a predictor for identifying people at high risk of falls.

Current research on the association between respiratory disease and risk of injury from falls has focused on chronic obstructive pulmonary disease (COPD), a common chronic bronchitis or emphysema disease. Milla’s study demonstrated the association between the number of chronic diseases and the risk of falls [27]. Jamas’ study reported an increased risk of falls in older people with chronic diseases [28]. A cross-sectional study by Lohman et al. [29] based on 9,258 community-dwelling older adults (≥60 years) found that the risk of injury from falls in older adults was primarily associated with COPD. This may be because COPD affects isometric knee extension, walking speed, single-leg posture, and limb balance in humans.

There is no conclusive evidence that neurological and cardiovascular diseases are risk factors for fall injury. Consistent with the results of this study, a cohort study in the USA showed that neurological disease (combined stroke and Parkinson’s disease) was not associated with the risk of injury from falls, and a case‒control study by Scott et al. [30] based on cross-sectional data found no association between having neurological disease and the risk of injury from falls, consistent with this study. A systematic review of cardiovascular disease and the risk of falls in people aged ≥50 years showed inconsistent associations between specific symptoms of cardiovascular disease and the risk of fall injury. The aetiological hypothesis between neurological disorders, cardiovascular disease, and fall injury needs to be further investigated in the future [31].

This study determined that the risk of damage from falls rose linearly with the number of chronic conditions in middle-aged and elderly individuals. Chronic diseases influence indices of physical function, somatic pain, general health, vitality, and mental health, and the greater the number of chronic diseases, the lower the overall health status and quality of life of older individuals. Cai et al.’s study [32] concluded that overall pain measures were associated with an increased risk of injury from falls in older people, which is generally consistent with the results of this study. The dose–response association between the number of chronic illnesses and the risk of injury from falls may be attributable to the cumulative or synergistic harm induced by several chronic conditions. Bhasin et al.’s study [33] included 4,050 female older adults (aged 60–79 years) and found a linear trend between the risk of fall injury and the prevalence of chronic disease, but no linear relationship was observed in this study among older adults aged ≥60 years. However, the cumulative effect of chronic disease prevalence on the risk of injury from falls was validated in this study [34].

Current research is less likely to discuss the impact of chronic disease interacting with other risk factors on the risk of fall injury. Gender, ADL, and history of fall injury are recognised risk factors for fall injury, and this study showed that women, middle-aged, and older people with chronic disease with impaired ADL and a history of fall injury had 1.67 (95% CI: 1.36–1.88), 1.68 (95% CI: 1.43–1.81), and 2.62 (95% CI: 2.51–2.94) times the rate of fall injury than men, middle-aged, and older people with intact ADL and never had a fall or chronic illness. 1.68 (95% CI: 1.43–1.81) and 2.62 (95% CI: 2.51–2.94) times more likely to have a history of falls. This finding is similar to the dose‒response relationship between chronic disease prevalence and fall injury risk, suggesting that the accumulation or synergy of risk factors significantly increases the risk of fall injury.

This study influence of chronic illness prevalence on the risk of fall injury in middle-aged and elderly individuals was investigated using a prospective cohort research design with a large sample size. A dose–response association was discovered between the prevalence of chronic illness and the risk of fall injury, giving solid evidence for the prevention and reduction of fall risk in middle-aged and older individuals. Follow-up studies will continue to study potential techniques for lowering the risk of falls among middle-aged and elderly adults, hence reducing the risk of falls in a variety of contexts for middle-aged and elderly individuals.

There are some limitations to this study in that information on fall injuries and chronic illnesses was obtained through self-reporting and may be subject to recall bias, but the use of a fall visit as a defining criterion for fall injuries in this study was able to reduce recall bias to a greater extent, and the use of illness diagnosed by a healthcare facility as a study factor made recall bias less likely and the results reliable. In addition, this study did not control for the influence of the home environment and social support factors.

## 5 Conclusion

This study reveals a dose–response relationship between chronic diseases and fall injuries in middle-aged and older adults. Prioritising fall prevention in middle age and managing chronic conditions can effectively reduce fall injuries and associated healthcare costs. The findings highlight the importance of early identification and management of chronic diseases to mitigate fall risks. Healthcare professionals should implement regular screenings, patient education, and comprehensive management plans tailored to this population’s unique needs. By addressing the complex interplay of chronic diseases and other risk factors, a proactive approach to fall prevention can be established, ultimately improving the quality of life for middle-aged and older adults.
